# Exercise as a multimodal intervention for adolescent depression: integrating neuroplasticity, BDNF signaling, stress biomarkers, exercise intensity, digital mental health interventions, and precision psychiatry

**DOI:** 10.3389/fpsyt.2026.1871297

**Published:** 2026-08-17

**Authors:** Jiqiao He, Shenggen Zhu, Jiahong Luo, Jun Ou

**Affiliations:** Physical Education Institute, Xinyu University, Xinyu, Jiangxi, China

**Keywords:** adolescents, BDNF signaling, digital mental health, exercise, exercise intensity, neuroepigenetic antidepressant, precision psychiatry, stress biomarkers

## Abstract

Adolescent depression constitutes an escalating global health issue, defined by complex interactions among biological, psychological, and social determinants. This narrative review synthesizes contemporary evidence on the effects of physical exercise as a non-pharmacological intervention for depressive symptoms in adolescents. Emerging empirical findings from randomized controlled trials suggest that exercise may provide mental health benefits across diverse adolescent populations, including individuals with depression and related comorbid conditions such as attention-deficit/hyperactivity disorder, obesity, and cardiometabolic disorders. Exercise interventions, particularly those involving moderate-to-vigorous intensity and multimodal approaches, have been associated with reductions in depressive symptoms, anxiety, and stress, while also improving quality of life, resilience, and cognitive performance. Mechanistically, these effects are thought to be partially mediated by exercise-induced neurobiological changes, including modulation of brain-derived neurotrophic factor, stress-related biomarkers such as cortisol, and myokine signaling pathways, which may support neuroplasticity and emotional regulation. However, direct evidence for epigenetic mechanisms in adolescent populations remains limited and should be interpreted with caution. Furthermore, digital mental health interventions integrated with physical activity may offer scalable preventive strategies within school-based and resource-limited settings. Nevertheless, substantial heterogeneity in outcomes persists, influenced by factors such as exercise intensity, adherence, baseline symptom severity, and individual genetic and environmental differences. While some studies report minimal or delayed effects, the overall evidence supports exercise as a promising complementary or preventive approach for adolescent depression. This review is a narrative synthesis, and future research should prioritize standardized methodologies, longitudinal designs, and mechanistic investigations to strengthen causal inference and translational applicability.

## 1 Introduction

Adolescent depression constitutes a significant and escalating global health concern, with major implications for mental, cognitive, and social functioning. It is among the leading contributors to disease burden in individuals aged 10–19 years, significantly increasing disability-adjusted life years and suicide risk (1, 2). Adolescence represents a unique developmental period characterized by ongoing maturation of prefrontal–limbic circuitry, heightened neuroplasticity, endocrine instability, and increased sensitivity to environmental stressors, which together distinguish adolescent depression from adult-onset forms in terms of neurobiological vulnerability and emotional regulation capacity (3). Despite increased awareness, many adolescents remain untreated or inadequately treated, reflecting persistent gaps in mental health care delivery (4). Although pharmacological and psychotherapeutic interventions are effective for some individuals, limitations including variable response, adverse effects, accessibility barriers, and partial targeting of lifestyle-related risk factors reduce their overall impact (5–8). These challenges highlight the need for scalable, low-risk, and multimodal and mechanistically informed adjunctive interventions.

Beyond traditional neurochemical models, adolescent depression can be conceptualized within an integrative mind–body framework, in which psychological and physiological systems are dynamically interconnected. Within this context, physical exercise may contribute to improvements in emotional regulation, self-efficacy, motivation, and social functioning, while depressive symptoms may simultaneously impair engagement in physical activity, highlighting a bidirectional relationship between mental state and behavioral capacity (9, 10). Exercise has been associated with antidepressant effects through modulation of multiple biological systems, including brain-derived neurotrophic factor (BDNF) signaling, hypothalamic–pituitary–adrenal (HPA) axis regulation, inflammatory processes, with putative and still-evolving involvement of epigenetic mechanisms (11, 12). However, evidence for exercise-related neuroepigenetic effects in adolescents remains preliminary, and findings across studies are heterogeneous due to variability in exercise modality, intensity, and duration (13, 14). Precision psychiatry refers to an emerging clinical framework that seeks to tailor interventions based on the integration of individual biological, psychological, and environmental characteristics. Within a biopsychosocial and mind–body framework, exercise should be conceptualized not only as a biological stimulus influencing pathways such as BDNF signaling, HPA axis regulation, and inflammatory processes, but also as a psychologically mediated intervention (15). Its effectiveness is closely linked to psychological factors including motivation, self-efficacy, emotional regulation, and subjective bodily experience (16), as well as social determinants such as family support, peer relationships, and broader environmental context (17). In adolescent populations, these psychological and relational dimensions are particularly critical, as they directly influence the feasibility, initiation, and maintenance of physical activity (18).

The present review synthesizes current evidence on exercise as a multimodal intervention for adolescent depression, focusing on neurobiological mechanisms, behavioral determinants, and emerging digital health approaches. It also situates these findings within the precision psychiatry framework, while acknowledging that its clinical application remains in an early and evolving stage. Methodological limitations, including small sample sizes, heterogeneous protocols, and reliance on peripheral biomarkers, are critically considered. Importantly, adolescents differ from adults in key neurobiological and developmental domains, including ongoing maturation of prefrontal–limbic circuits, higher neuroplastic potential, greater hormonal variability, and increased sensitivity to environmental influences, which may alter both vulnerability to depression and responsiveness to exercise-based interventions. Overall, exercise may represent a promising complementary strategy for adolescent depression; however, further standardized, longitudinal, and mechanistically robust but cautiously interpreted research is required to strengthen causal inference and clinical translation.

## 2 Epidemiology and clinical complexity of adolescent depression

Adolescent depression constitutes a heterogeneous and multifactorial disorder that manifests during a critical developmental phase characterized by significant biological, psychological, and social transformations. Worldwide, prevalence estimates indicate that approximately 10–20% of adolescent’s experience clinically significant depressive symptoms, with major depressive disorder impacting a considerable subset of this demographic (19, 20). Importantly, the incidence of depression escalates markedly during early to mid-adolescence, particularly among females, reflecting the complex interplay of pubertal hormonal fluctuations, neurodevelopmental changes, and gender-specific psychosocial stressors (21). The vulnerability experienced in early life is further exacerbated by adverse childhood experiences, which encompass trauma, neglect, and familial dysfunction, all of which can have enduring impacts on stress-response mechanisms and emotional regulation (22). Importantly, these developmental and environmental exposures are increasingly understood within a neurodevelopmental–inflammatory framework, in which chronic low-grade inflammation interacts with ongoing maturation of prefrontal–limbic circuitry, thereby increasing vulnerability to affective dysregulation during adolescence (23, 24). Within this context, physical exercise has been proposed as a potential modulator of inflammatory activity and neurodevelopmental plasticity, suggesting a mechanistic link between lifestyle factors and adolescent mental health outcomes (25, 26). These initial exposures are increasingly recognized as influencing neurobiological pathways and heightening the risk of depression in later stages of life. The intricate clinical nature of adolescent depression is emphasized by its elevated prevalence of comorbidity with both psychiatric and somatic disorders. Attention-deficit/hyperactivity disorder (ADHD) represents one of the most frequently co-occurring neurodevelopmental disorders, characterized by overlapping attributes such as emotional dysregulation and compromised executive functioning (27). From an intervention perspective, ADHD may also reduce responsiveness to structured physical activity due to deficits in attention, reward processing, and executive functioning, thereby influencing adherence to exercise-based interventions (28). The existence of ADHD complicates the diagnostic process and correlates with an earlier onset and heightened severity of depressive symptomatology. In a similar vein, obesity has emerged as a prominent comorbid condition, with bidirectional associations that connect depressive symptoms to increased adiposity, reduced physical activity, and psychosocial stressors (29). Obesity-related chronic low-grade inflammation may further modify neurobiological responsiveness to exercise interventions, potentially influencing antidepressant-related effects of physical activity (25, 30).

Adolescents experiencing obesity frequently encounter stigma and diminished self-esteem, which may further intensify their depressive symptomatology. Beyond the scope of obesity, cardiometabolic anomalies, encompassing insulin resistance and dyslipidemia, are progressively acknowledged in adolescent populations afflicted with depression, indicating that affective disorders may possess systemic physiological ramifications even at a nascent developmental stage (31). These overlapping biological pathways are relevant for exercise-based interventions, as physical activity has been shown to modulate both inflammatory signaling and stress-response systems (25, 26). Shared pathophysiological mechanisms, such as persistent low-grade inflammation and the dysregulation of stress-response systems, particularly the HPA axis, are hypothesized to establish a link between depression and metabolic dysfunction (23). These comorbid conditions exacerbate the overall disease burden and complicate treatment modalities, especially when pharmacological interventions pose potential detriments to metabolic health. Psychosocial and environmental determinants are pivotal in the initiation and progression of adolescent depression. Familial factors, including parental psychopathology, familial conflict, and the absence of emotional support, are consistently correlated with an elevated risk (32). Peer influences are of paramount importance during the developmental stage of adolescence, with phenomena such as bullying and social exclusion significantly exacerbating the manifestation of depressive symptoms (33). Furthermore, the pressures associated with academic performance and stress serve as critical catalysts, particularly within highly competitive educational environments. The escalating impact of digital platforms, particularly the utilization of social media, has introduced an additional layer of complexity, with empirical evidence correlating excessive engagement with adverse mental health outcomes via mechanisms such as disrupted sleep patterns and detrimental social comparisons (34). Excessive screen time is also associated with sedentary behavior, which may reduce engagement in physical activity. Conversely, exercise-based interventions may counteract these effects through behavioral activation and reward system engagement (35). Socioeconomic disadvantage further intensifies vulnerability, given that adolescents from economically disadvantaged backgrounds frequently encounter a confluence of stressors, diminished access to mental health resources, and limited opportunities for engaging in supportive activities such as organized physical exercise (36). These intertwined biological, psychological, and environmental factors elucidate the multifaceted nature of adolescent depression and emphasize the necessity for integrative approaches that concurrently address both mental and physical health within broader societal contexts. Importantly, sex differences, obesity status, and neurodevelopmental conditions such as ADHD may further moderate vulnerability to depression and response to exercise-based interventions, underscoring the importance of stratified and precision-based approaches (37, 38).

## 3 Exercise as a multimodal intervention in adolescents

### 3.1 Types of exercise interventions

Exercise interventions targeting adolescents encompass a variety of modalities, including aerobic training, resistance training, high-intensity interval training (HIIT), and mind–body practices such as yoga. Aerobic exercise represents the most extensively researched modality and is consistently correlated with decreases in depressive symptoms, likely attributable to its influence on cardiovascular fitness and neurobiological regulation (39). Resistance training has also demonstrated potential benefits, particularly in enhancing self-esteem and physical self-concept, constructs that are intricately linked to mood (40). Emerging research indicates that HIIT may facilitate rapid neurotrophic and metabolic adaptations, providing time-efficient advantages for adolescents exhibiting low adherence (41). However, the current evidence base for HIIT in adolescent depression remains less extensive and more heterogeneous compared to traditional aerobic exercise, limiting the ability to draw definitive conclusions regarding its comparative efficacy. Concurrently, mind–body interventions may mitigate stress and improve emotional regulation through parasympathetic activation (42). Nevertheless, the variability in intervention design and the absence of standardization impede direct comparisons among studies. Overall, aerobic exercise currently has the strongest and most consistent evidence base, while HIIT and resistance training are considered promising but still emerging modalities. A multimodal approach that integrates various exercise modalities may yield synergistic benefits, customized to individual preferences and clinical profiles.

### 3.2 Exercise intensity, frequency, and dose–response

The antidepressant effects of physical exercise among adolescents appear to be significantly moderated by the parameters of intensity, frequency, and total training volume, although the optimal configurations continue to be a subject of scholarly discourse. Exercise characterized by moderate-to-vigorous intensity is typically correlated with more substantial ameliorations in depressive symptomatology in comparison to low-intensity engagement, potentially attributable to an augmented release of neurotrophic factors such as BDNF and enhanced regulation of the HPA axis (43, 44). Frequency is also a pivotal element, with the most efficacious interventions typically comprising at least three sessions per week across a duration of 8 to 12 weeks (45). Nonetheless, excessive intensity or training volume may precipitate fatigue, diminished adherence, or even negative psychological outcomes in young people (46). Importantly, the current literature does not provide a fully standardized or universally accepted exercise prescription for adolescent depression, and substantial heterogeneity exists across studies in terms of modality, intensity, duration, and outcome measures. Dose-response relationships are likely to exhibit non-linear characteristics, indicating the necessity for individualized exercise prescriptions. Current empirical evidence underscores the significance of achieving an equilibrium between physiological advantages and enjoyment, as well as sustainability, to optimize long-term mental health outcomes.

### 3.3 Effects on mental health outcomes

Engagement in physical exercise yields extensive and clinically significant impacts on mental health outcomes among adolescents, transcending mere reductions in depressive symptomatology. Consistent participation in physical activities has been correlated with enhancements in anxiety levels, self-esteem, cognitive functioning, and overall psychological welfare (47). The underlying mechanisms for these advantageous effects are attributed to augmented neuroplasticity, elevated levels of BDNF, diminished inflammatory responses, and enhanced resilience to stress (11). Furthermore, physical exercise may facilitate social interactions and foster a sense of achievement, both of which are critical during the developmental stage of adolescence (48). Notably, certain research indicates that exercise interventions can attain efficacy levels comparable to those of pharmacological or psychotherapeutic modalities in cases of mild-to-moderate depression (49). However, this conclusion should be interpreted cautiously, as evidence quality varies considerably across study designs, and direct head-to-head comparisons with pharmacological or psychotherapeutic interventions remain limited. Importantly, the effects of exercise on mental health outcomes should be interpreted within distinct clinical and preventive contexts, as evidence varies substantially across intervention types.

#### 3.3.1 Preventive context

In prevention-focused studies, particularly school- or community-based interventions, physical activity has been shown to reduce the risk of developing depressive symptoms and improve overall psychological well-being in general adolescent populations (50, 51).

#### 3.3.2 Adjunctive context

In adjunctive settings, exercise is most frequently examined as a complementary intervention alongside psychotherapy or pharmacological treatment, where it may enhance symptom reduction and improve functional outcomes (49, 52).

#### 3.3.3 Treatment context

In clinical treatment trials involving adolescents with diagnosed depressive disorders, exercise demonstrates beneficial effects; however, the evidence base remains heterogeneous, and effect sizes vary depending on severity, adherence, and intervention design. Notably, certain research indicates that exercise interventions may demonstrate beneficial effects comparable to standard care in cases of mild-to-moderate depression; however, direct equivalence with pharmacological or psychotherapeutic treatments cannot be conclusively established due to limited head-to-head trials and variability in study quality (48, 53). Nevertheless, the heterogeneity in study quality and the variability in outcome measures present challenges for interpretation. In spite of these constraints, the aggregate body of evidence substantiates the role of exercise as a significant adjunctive or independent intervention within the realm of adolescent mental health care.

## 4 Neurobiological mechanisms underlying exercise effects

### 4.1 Integrated neurobiological and epigenetic mechanisms of exercise in adolescent depression

#### 4.1.1 Evidence from clinical trials on exercise-based interventions for adolescent depression and mental health outcomes

Overall, randomized controlled trials and school-based interventions collectively suggest that exercise and physical activity–based interventions are associated with improvements in depressive symptoms, anxiety, stress regulation, and broader psychological functioning in adolescents. However, the evidence is heterogeneous with respect to intervention modality, duration, and population characteristics, indicating variability in effect sizes and sustainability of outcomes (**Table 1**). In particular, early digital and school-based mental health interventions demonstrate that structured behavioral and psychoeducational programs can significantly reduce depressive and anxiety symptoms, supporting the broader effectiveness of integrated mental health strategies in adolescent populations with limited access to care (54). Across school-based and community settings, multicomponent interventions integrating structured physical activity, health education, and psychosocial components consistently demonstrate improvements in depressive symptoms and well-being, suggesting that combined behavioral approaches may be more effective than isolated interventions (59, 62, 67). These findings support the view that exercise-related mental health benefits are not limited to neurobiological mechanisms alone but are also mediated through behavioral activation, social engagement, and improved health-related behaviors. In parallel, exercise interventions in adolescents show differential effects depending on intensity and modality. Aerobic and structured moderate-to-vigorous physical activity interventions are generally associated with more consistent reductions in depressive symptoms and improvements in self-esteem and psychological functioning (55, 69). In contrast, interventions with shorter duration or lower frequency often demonstrate weaker or non-significant effects, highlighting the importance of sufficient exposure and training dose (60, 61).

**Table 1:** Summary of randomized controlled and intervention studies on exercise- and lifestyle-based programs and their effects on mental and physical health outcomes in adolescents.

Population	Intervention	Duration	Outcomes	Findings	Ref
390 Chinese high school students	Culturally adapted digital mental health literacy + CBT-based program	12 weeks	PHQ-A (depression), GAD-7 (anxiety)	Significant reduction in anxiety (-2.54, P<0.001) and depression (-1.91, P<0.001); scalable school-based prevention strategy	(54)
266 adolescents (eighth-grade students)	Low-, moderate-, and vigorous-intensity physical education	12 weeks	CES-D, muscle strength (long jump, grip strength)	Vigorous intensity showed greatest reduction in depressive symptoms; lower-limb strength improvements predicted depression reduction	(55)
82 adolescents with ADHD	Moderate-to-vigorous physical exercise (PE program)	3 weeks + 3-month follow-up	Stress, anxiety, depression, cortisol	Reduced stress and altered cortisol levels post-intervention; effects not sustained at follow-up	(56)
110 obese, hypertensive adolescents	Moderate/low-intensity exercise + individualized nutrition vs routine care	6 weeks	SCARED, CDI, PedsQL, BMI, BP	Significant improvements in anxiety, depression, BMI, BP, and quality of life; combined intervention superior to control	(57)
88 adolescents with ADHD	Aerobic exercise (60 min/week) vs control	12 weeks + 3-month follow-up	Depression, anxiety, stress, resilience, inhibitory control	Reduced depression, anxiety, stress; improved cognitive control and resilience (partial sustainability at follow-up)	(58)
524 high school students	Combined physical activity + anti-stigma education	3 months	Depressive symptoms, physical activity, weight stigma, weight loss behaviors	Reduced depressive symptoms and weight stigma; increased physical activity; no change in weight loss	(59)
306 adolescents	Multicomponent physical activity (strength + cardio + lifestyle education) vs control	12 weeks	Anxiety, depression, sleep, psychological well-being	Significant benefits only in adolescents with moderate/severe symptoms; reduced anxiety and depression; preserved well-being vs decline in controls	(60)
207 adolescents & young adults with ADHD	Exercise intervention vs bright light therapy vs treatment-as-usual (mHealth delivery)	10 weeks	Depressive symptoms (observer-rated)	Small reductions in depressive symptoms in exercise group; no significant between-group differences; very low adherence and feasibility issues	(61)
320 adolescents (cluster RCT)	Structured school-based physical activity + education vs control	12 weeks	Depression, life satisfaction	Significant reduction in depressive symptoms and increased life satisfaction in intervention group compared to controls	(62)
779 high school adolescents	CBT-based lifestyle intervention + physical activity + nutrition education	15 weeks + 12-month follow-up	BMI, depressive symptoms	Long-term reductions in BMI and depressive symptoms; strongest effects in adolescents with elevated baseline depression	(63)
87 adolescents receiving depression treatment	Preferred-intensity aerobic exercise + treatment-as-usual vs TAU alone	12 sessions + 6-month follow-up	CDI-2, quality of life, physical activity	No immediate effect post-intervention, but significant depressive symptom reduction at 6-month follow-up	(64)
26 adolescents with depression	Preferred-intensity exercise intervention (qualitative evaluation)	Intervention period + post-interview	Lived experience, mood, motivation	Participants reported improved mood, enjoyment, achievement, and psychosocial benefits beyond symptom reduction	(65)
56 adolescents with congenital heart disease (ToF/Fontan)	Standardized exercise training vs care-as-usual	12 weeks	HRQoL, social functioning, parental mental health	Exercise effects on HRQoL were moderated by parental mental health; poorer parental mental health associated with reduced adolescent social functioning improvements	(66)
19 Hispanic adolescents (pilot RCT, school-based)	Cognitive-behavioral + physical activity + nutrition (COPE TEEN) vs attention control	15 weeks	Depression, anxiety, lifestyle behaviors, metabolic markers	Improved depressive and anxiety symptoms, healthier lifestyle behaviors, and improved lipid profiles in overweight adolescents	(67)
Obese adolescents (n=81)	Supervised exercise therapy	14 weeks	Depression, self-esteem, BMI	Improved psychological outcomes; no BMI change	(68)
Low-income children (n=66)	Aerobic fitness program	6 weeks	Depression, anxiety, self-esteem	Reduced depression; increased self-esteem	(69)

Abbreviations used in this table: n, sample size; RCT, randomized controlled trial; ADHD, attention-deficit/hyperactivity disorder; CBT, cognitive behavioral therapy; PA, physical activity; BMI, body mass index; BP, blood pressure; QoL, quality of life; HRQoL, health-related quality of life; CES-D, Center for Epidemiologic Studies Depression Scale; PHQ-A, Patient Health Questionnaire for Adolescents; GAD-7, Generalized Anxiety Disorder-7; CDI, Children’s Depression Inventory; SCARED, Screen for Child Anxiety Related Emotional Disorders; BLT, bright light therapy; m-health, mobile health; SD, standard deviation.

From a mechanistic perspective, improvements in mental health outcomes are frequently accompanied by changes in physical fitness, metabolic regulation, and stress-related biomarkers, suggesting interconnected physiological and psychological pathways. For example, increases in muscular fitness and aerobic capacity have been linked with reductions in depressive symptom severity, indicating a potential dose–response relationship between physical conditioning and mood regulation (55). Similarly, combined lifestyle interventions targeting both physical activity and nutrition appear to produce broader improvements in psychological well-being and cardiometabolic health in high-risk adolescents (57). Longitudinal evidence further indicates that psychosocial and family-related factors may influence the sustainability and magnitude of exercise-related mental health benefits. For instance, parental mental health status has been shown to moderate the impact of exercise interventions on adolescents’ social functioning, highlighting the importance of relational and environmental context in shaping outcomes (66). Importantly, longitudinal and lifestyle-based intervention studies indicate that improvements in depressive symptoms may persist over time and are influenced by broader behavioral health changes, including physical fitness, weight status, and health-related quality of life (63).

Several trials highlight that baseline clinical and psychosocial characteristics significantly influence intervention outcomes. Adolescents with comorbid conditions such as ADHD, obesity, or cardiometabolic risk factors may exhibit different response trajectories, emphasizing the need to interpret findings within a comorbidity-informed framework rather than attributing effects solely to primary depressive symptomatology (56–58, 68). In such populations, improvements may extend beyond mood symptoms to include executive functioning, self-esteem, and physical self-perception. Notably, while most studies report short-term improvements in depressive symptoms and related outcomes, long-term sustainability of effects is less consistent. Some interventions demonstrate delayed or follow-up benefits, suggesting that psychological and behavioral adaptations may emerge over time rather than immediately post-intervention (64). Moreover, participant-reported outcomes indicate that autonomy, enjoyment, and perceived self-efficacy are critical mediators of adherence and psychological benefit, underscoring the importance of motivational and experiential factors in exercise-based interventions (65). Finally, while some trials report significant reductions in depressive symptoms and anxiety, others show null findings, particularly in studies with brief duration or low adherence, reinforcing the importance of intervention dose, structure, and engagement as key moderating factors (61, 68). Collectively, the evidence supports exercise as a promising but context-dependent intervention for adolescent mental health, with effects influenced by biological, behavioral, and psychosocial mechanisms rather than a uniform treatment response.

#### 4.1.2 Molecular mechanisms linking exercise to antidepressant effects in adolescents

Overall, physical exercise is associated with multiple converging molecular pathways that are proposed to contribute to antidepressant effects observed in adolescents. A key mechanism involves the upregulation of BDNF, which may support synaptic plasticity, neurogenesis, and executive functioning, particularly in response to moderate-to-vigorous aerobic and resistance training. However, it is important to note that much of the mechanistic evidence in adolescents is indirect, and causal relationships remain insufficiently established. In parallel, physical activity is associated with modulation of the HPA axis, which may contribute to more adaptive cortisol regulation. This effect has been particularly explored in populations with neurodevelopmental or clinical vulnerability, such as ADHD, although findings remain heterogeneous and context-dependent. Similarly, exercise is linked to reductions in peripheral inflammatory markers, including IL-6 and TNF-α, suggesting a potential pathway connecting immune regulation with mood-related outcomes. Nevertheless, most evidence is derived from peripheral biomarkers rather than direct central nervous system measurements in adolescents. Exercise has also been suggested to influence monoaminergic signaling systems, including serotonin, dopamine, and norepinephrine pathways, which are involved in reward processing and emotional regulation. However, these effects are largely inferred from adult and preclinical studies, and direct validation in adolescent populations remains limited. With respect to epigenetic regulation, emerging evidence indicates that physical activity may induce epigenetic modifications, including DNA methylation changes and alterations in microRNA expression. These mechanisms are considered promising for explaining long-term neurobiological adaptations; however, the current evidence base in adolescents is preliminary, and most findings originate from indirect human evidence or animal models. Therefore, the role of epigenetic mechanisms in exercise-related antidepressant effects in adolescents should be interpreted as hypothesis-generating rather than confirmatory. In addition, exercise-related improvements in metabolic and cardiometabolic function, including insulin sensitivity and lipid regulation, may indirectly support cognitive performance and emotional regulation. These systemic effects highlight the broader physiological relevance of physical activity beyond central neurobiological pathways. Importantly, the current mechanistic literature is characterized by several limitations. First, direct causal evidence in adolescent populations is scarce, and most conclusions are based on correlational findings or extrapolation from adult or animal research. Second, many observed biological changes are derived from peripheral biomarkers, which may not fully reflect central nervous system processes. Third, heterogeneity in exercise modality, intensity, and duration further limits mechanistic comparability across studies. Overall, while these molecular pathways provide a coherent and biologically plausible framework for understanding the antidepressant effects of exercise, they should be interpreted cautiously. The available evidence supports associative and mechanistic hypotheses, rather than definitive causal mechanisms, particularly within adolescent populations.

#### 4.1.3 Exercise-based interventions for adolescent depression: methodological considerations and limitations

The studies reviewed indicate an overall favorable impact of exercise on depressive symptoms and related outcomes; however, methodological limitations reduce the strength and certainty of the evidence. As this work is a narrative review, no formal systematic risk-of-bias tool (e.g., RoB-2) or evidence grading framework (e.g., GRADE) was applied. Instead, a qualitative methodological appraisal was conducted based on study design characteristics and reporting quality. Sample sizes varied considerably, ranging from small pilot studies (n=19–26) to larger cluster randomized trials (>500 participants), limiting statistical power and external validity. Intervention duration was predominantly short-term (3–12 weeks), with only a minority extending beyond 12 months, restricting conclusions regarding long-term efficacy. Numerous studies lacked adequate blinding, increasing the risk of performance and detection bias, particularly in trials relying on self-reported depressive symptom measures. In addition, reliance on self-report outcomes introduces potential measurement bias, which may overestimate intervention effects. Adherence to exercise interventions was inconsistently reported and often suboptimal, especially in digital or mobile health-based interventions, introducing potential attrition bias. Publication bias may also be present, as studies reporting positive outcomes are more likely to be published than null or negative findings. Small-study effects further limit the robustness of pooled interpretations; as smaller trials tend to report larger effect sizes. The heterogeneity in exercise modalities, intensity, and outcome measures further limits comparability across studies. Additionally, several trials focused on specific subpopulations (e.g., ADHD, obesity), which may reduce generalizability to broader adolescent populations. In summary, although findings are promising, the current evidence base remains methodologically heterogeneous. Future research should prioritize standardized study designs, improved reporting transparency, and larger well-controlled trials to strengthen the evidence base.

A critical translational limitation in interpreting neurobiological findings in adolescent depression relates to the use of peripheral biomarkers as indirect indicators of central nervous system processes. Although biomarkers such as inflammatory cytokines and stress hormones provide valuable insights into systemic physiological states, their relationship with central neurobiological activity is not always direct or consistent. Recent literature describing diagnostic discordance between radiological, biomarker, and pathological findings in complex CNS disorders further highlights the limitations of relying solely on peripheral biomarkers to infer central neurobiological mechanisms (70).

### 4.2 BDNF and neuroplasticity

#### 4.2.1 Evidence from clinical trials on exercise-based interventions for neuroplasticity in adolescent

A randomized controlled trial examined the effects of a 9-week aerobic training regimen on cardiorespiratory fitness, cognitive function, and BDNF in adolescents. A total of 121 participants were recruited, with 85 completing the study. Participants were allocated to high-intensity training, moderate-intensity training, or control conditions. Findings showed significant improvements in VO_2_max in the high-intensity group; however, no statistically significant changes were observed in inhibitory control or plasma BDNF levels across groups (71) (**Table 2**). A non-significant trend toward improved cognitive accuracy was observed in high-fitness individuals. Williams et al. reported similar findings, where a 60-minute football intervention did not significantly alter cognitive performance or BDNF levels overall, although physical fitness was associated with faster reaction times and moderated cognitive responsiveness in high-fit adolescents (72). Roh et al. reported increases in serum BDNF following a 16-week Taekwondo intervention in overweight adolescents; however, these changes occurred alongside broader metabolic improvements, making it difficult to isolate BDNF-specific effects (73). In a larger randomized controlled trial, exercise combined with dietary intervention showed no significant between-group differences in BDNF, although within-group associations suggested relationships between BDNF changes and metabolic markers such as fasting glucose (74). Similarly, Goldfield et al. found no significant changes in resting peripheral BDNF across different exercise modalities, suggesting that exercise-induced neurotrophic responses may not consistently manifest in peripheral circulation in adolescents (75).

**Table 2:** Exercise interventions and BDNF-related outcomes, and neurocognitive outcomes in adolescents.

Population	Intervention	Duration	Outcomes	Findings	Ref
Danish adolescents (n=121; 85 completed)	High- vs moderate-intensity aerobic training (cycling + running) vs control	9 weeks	VO_2_max, inhibitory control, plasma BDNF	VO_2_max increased in intensity-dependent manner; no significant change in resting BDNF; slight improvement trend in inhibitory control accuracy	(71)
Adolescents (n=36)	Acute football activity vs seated rest (crossover design)	Single 60-min session + follow-up measures	Information processing, working memory, inhibitory control, circulating BDNF	No overall cognitive or BDNF differences vs rest; higher fitness improved cognitive performance and enhanced post-exercise working memory; BDNF unchanged	(72)
Overweight/obese adolescents (n=20)	Taekwondo training (5×/week) vs control	16 weeks	Oxidative stress markers, myokines, BDNF, fitness	Increased BDNF and SOD; reduced MDA; improved physical fitness and body composition	(73)
Adolescents with obesity (n=202)	Aerobic + resistance training vs control (diet standardization in all groups)	6 months	BDNF, fasting glucose, insulin, HOMA-IR, HbA1c	No between-group differences in BDNF; within exercise group, increased BDNF associated with improved glucose metabolism and insulin sensitivity	(74)
Adolescents with overweight/obesity (n=304)	Aerobic vs resistance vs combined training vs control	22 weeks	Resting serum BDNF	No significant changes in BDNF across any intervention group	(75)
Children (n=318)	High-intensity training vs active control	6 weeks	Executive function, working memory, BDNF-related genotype effects	Improved cognitive control and working memory; effects moderated by BDNF Val66Met genotype	(76)
Children with overweight/obesity (n=101)	Combined aerobic exercise program vs control	20 weeks	Cognitive flexibility, academic skills, hippocampal volume, BDNF-related genetic score	Cognitive improvements only in genetically “favorable” group; BDNF-related genotype moderated exercise response	(77)
Children with dysgraphia (n=40)	Motor exercise training (Lincoln-Oseretsky-based) vs control	12 weeks	Serum BDNF, executive function (WCST)	Significant increases in BDNF and executive function; strong association between BDNF and cognition	(78)
Elementary students (n=30)	Taekwondo-based combined exercise vs control	12 weeks	BDNF, NGF, working memory, physical fitness	Improved NGF and physical fitness; no significant change in BDNF or working memory	(79)
Healthy children (n=30)	Taekwondo training vs control	16 weeks	BDNF, VEGF, IGF-1, cerebral blood flow, cognitive function	Increased BDNF and neurotrophic factors; improved cognitive function (Stroop test); no change in cerebral blood flow	(80)

n, sample size; RCT, randomized controlled trial; BDNF, brain-derived neurotrophic factor; NGF, nerve growth factor; VEGF, vascular endothelial growth factor; IGF-1, insulin-like growth factor-1; VO_2_max, maximal oxygen uptake; WCST, Wisconsin Card Sorting Test; RPE, rating of perceived exertion; TKD, Taekwondo; HIT, high-intensity training; MIT, moderate-intensity training; CON, control group; HRR, heart rate reserve; MCAs, middle cerebral artery peak systolic velocity; MCAd, middle cerebral artery end diastolic velocity; MCAm, mean cerebral blood flow velocity; PI, pulsatility index; and CI, confidence interval.

Moreau et al. demonstrated improvements in cognitive control and working memory following HIIT in children; however, these effects were moderated by genetic factors (BDNF Val66Met polymorphism), indicating inter-individual variability rather than uniform neurotrophic effects (76). Importantly, these findings relate to pediatric cognitive development and should be interpreted cautiously in relation to adolescent depression specifically. Plaza-Florido et al. similarly reported that cognitive improvements following exercise were dependent on genetic profiles, further supporting the role of individual variability in neurocognitive responsiveness rather than direct causal BDNF-mediated effects (77). Ghafori et al. reported increases in serum BDNF and executive function following motor exercise in children with dysgraphia, although the clinical relevance to depressive pathology remains indirect (78). Kim et al. observed increases in NGF but only non-significant changes in BDNF following a 12-week exercise intervention, further highlighting inconsistency in peripheral BDNF responses across pediatric populations (79). Cho et al. reported increases in BDNF alongside other neurotrophic factors (VEGF, IGF-1), suggesting that neuroplastic adaptations may involve broader molecular networks rather than isolated BDNF effects (80).

#### 4.2.2 Molecular pathways mediating exercise-induced neuroplasticity in adolescents

**Figure 1:**
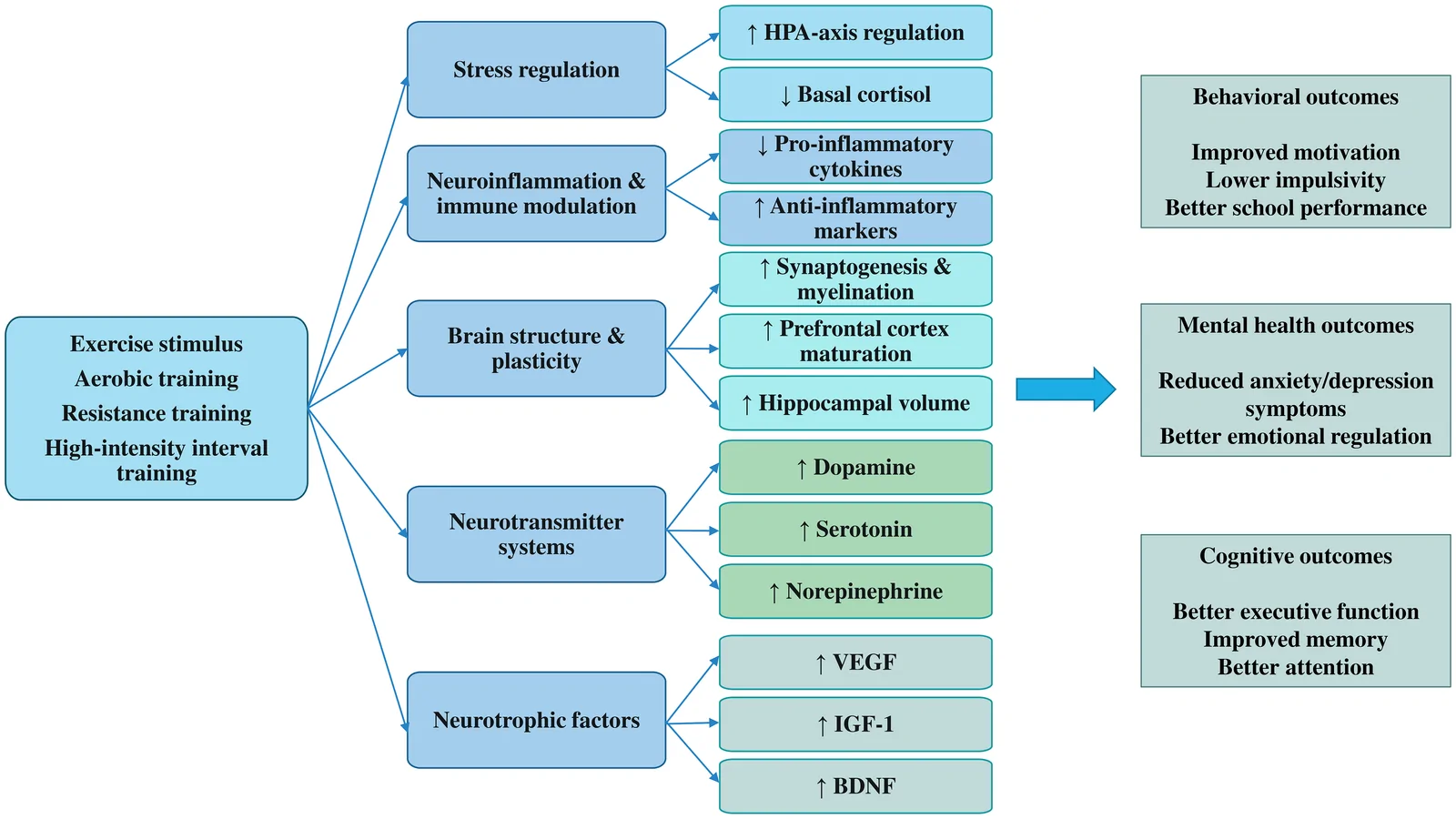
Neurobiological mechanisms underlying the effects of exercise in adolescents. This conceptual diagram illustrates how different forms of exercise (aerobic, resistance, high-intensity interval training, and motor-skill–based activities) activate multiple neurobiological pathways that shape adolescent brain function and behavior. Exercise increases neurotrophic factors such as BDNF, IGF-1, and VEGF, enhances monoaminergic neurotransmission (dopamine, serotonin, norepinephrine), and promotes structural brain adaptations including hippocampal and prefrontal cortex development, synaptogenesis, and myelination. Concurrently, exercise reduces pro-inflammatory cytokines and improves HPA-axis regulation, lowering stress-related cortisol responses. Together, these mechanisms contribute to improved cognitive performance, better emotional regulation, reduced symptoms of anxiety and depression, and enhanced behavioral functioning during adolescence.

Physical activity influences multiple molecular pathways associated with neuroplasticity, including BDNF, NGF, VEGF, and IGF-1 signaling. However, evidence for consistent peripheral BDNF elevation in adolescents remains mixed, and several trials report null findings despite cognitive improvements, suggesting a possible dissociation between peripheral biomarkers and central neurobiological processes. Importantly, serum or plasma BDNF should be interpreted cautiously as a surrogate marker of central BDNF activity, as peripheral levels may be influenced by non-neuronal sources and do not necessarily reflect brain-derived neurotrophic signaling. Moreover, observed associations between BDNF and cognitive outcomes are largely correlational, and causal interpretations cannot be firmly established based on current adolescent evidence. Exercise-related improvements in cognition and executive function are likely mediated by integrated neurotrophic, metabolic, and vascular mechanisms rather than a single BDNF-dependent pathway. Additionally, variability in outcomes is influenced by exercise intensity, duration, developmental stage, and genetic polymorphisms. Overall, while neurotrophic signaling provides a plausible framework for understanding exercise-related brain effects, the current evidence base in adolescents supports associative and multicausal interpretations rather than direct causal BDNF-driven mechanisms (**Figure 1**).

#### 4.2.3 Critical appraisal of exercise–brain health trials in children and adolescents

The available pediatric exercise trials provide generally supportive but methodologically variable evidence regarding neurocognitive and neurobiological outcomes. Across studies, sample sizes range from small pilot investigations to moderately sized randomized trials, which may limit statistical power, particularly in mechanistic and subgroup analyses. Intervention duration is also highly heterogeneous, typically spanning from several weeks to a few months, which may be insufficient to evaluate longer-term developmental or neuroplastic processes associated with sustained behavioral and physiological adaptation. A further limitation relates to the frequent reliance on peripheral biomarkers, such as serum or plasma BDNF, which may not fully capture central nervous system activity and therefore should be interpreted cautiously as indirect indicators of brain-related changes. In addition, variability in cognitive and behavioral outcome measures across studies limits cross-study comparability. Methodological concerns are also present in several trials, including incomplete blinding procedures, reliance on self-reported cognitive or behavioral outcomes, and inconsistent reporting of intervention adherence. Although emerging studies exploring genetic or biological stratification are promising, most remain exploratory and underpowered for robust subgroup inference. Overall, while pediatric exercise interventions appear promising for brain health and neurocognitive outcomes, the current evidence base is characterized by methodological heterogeneity and limited mechanistic resolution. These limitations highlight the need for well-powered, developmentally informed, and standardized longitudinal studies to better clarify the neurobiological and cognitive effects of exercise during childhood and adolescence.

## 5 Barriers, adherence, and real-world challenges

Despite the extensively documented advantages of physical activity in enhancing mental health outcomes among adolescents, the practical application of such interventions remains fraught with difficulties, with adherence emerging as a significant barrier. A considerable number of adolescents, particularly those grappling with depressive disorders or co-occurring conditions, exhibit low levels of engagement and elevated dropout rates within structured exercise programs. Importantly, psychological readiness represents a critical prerequisite for engagement in exercise-based interventions, as diminished adherence is frequently driven by core symptoms of depression, such as fatigue, anhedonia, diminished motivation, and compromised executive functioning, which collectively obstruct the initiation and sustainability of physical activity. Furthermore, psychosocial variables, including academic pressures, insufficient social support, concerns regarding body image, and the presence of stigma, further exacerbate the withdrawal from exercise interventions. In addition, psychological and relational factors such as low self-efficacy, emotional dysregulation, body shame, and social withdrawal may further reduce the capacity to initiate and sustain physical activity unless appropriately addressed through supportive interventions. Environmental and structural impediments also exert a considerable influence, including restricted access to safe recreational environments, inadequate infrastructural support within educational institutions, and temporal limitations embedded within academic schedules, all of which can hinder opportunities for regular physical engagement. Furthermore, interventions that are excessively homogenized or neglect to consider individual preferences such as exercise intensity, modality, and social context, may diminish participation and long-term viability. Empirical evidence indicates that inflexible, high-intensity programs lacking sufficient customization can result in suboptimal adherence, whereas interventions that resonate with adolescents’ preferences are more likely to promote enhanced compliance and favorable psychological outcomes. In response to these challenges, novel strategies underscore the critical significance of personalization, adaptability, and seamless integration into everyday life. However, beyond personalization alone, successful implementation often requires psychological support, motivational enhancement strategies, family involvement, and broader relational scaffolding to enhance readiness and sustain engagement. Customizing exercise regimens to align with individual capabilities and preferences, incorporating pleasurable and socially interactive activities, and embedding programs within educational environments can further significantly enhance participation rates when these supportive elements are in place. The utilization of digital health technologies, such as mobile applications and wearable devices, provides further avenues to monitor progress, furnish feedback, and bolster motivation. Significantly, it is imperative to acknowledge that digital interventions are concomitantly linked with notable limitations that necessitate examination from the perspective of implementation science. These limitations encompass screen fatigue, which has the potential to diminish prolonged engagement over an extended period (81); inequitable access to digital resources, which may exacerbate socioeconomic disparities in the reach of interventions (82); concerns regarding privacy and data security, particularly in applications focusing on adolescent mental health (83); and the phenomenon of adherence decay over time, characterized by a progressive decline in initial engagement levels during the sustained utilization of digital health interventions (84). From the vantage point of implementation science, the effective translation of exercise-based and digital interventions into tangible adolescent environments necessitates a thorough consideration of scalability, acceptability, feasibility, and sustainability (85). Empirical evidence indicates that, in the absence of structured implementation frameworks, even interventions that are demonstrably effective may encounter failure due to contextual impediments and diminishing engagement trajectories over time. Behavioral strategies, including goal-setting, motivational interviewing, and peer support networks, may further augment adherence levels. In summation, surmounting these obstacles necessitates a comprehensive approach that amalgamates biological understanding with behavioral and environmental factors to facilitate the effective implementation of exercise interventions within real-world adolescent populations. Crucially, psychological readiness should be considered a foundational prerequisite for engagement, particularly in clinically depressed adolescents, and must be actively supported through integrated psychological and social interventions rather than assumed at baseline.

## 6 Clinical and translational implications

The expanding corpus of research situates physical exercise as a clinically pertinent intervention for adolescent depression, potentially serving as both a supplementary measure and, in particular instances, a primary therapeutic approach. Although pharmacological treatment and psychotherapy are considered the gold standard interventions for moderate to severe depression, exercise presents a biologically substantiated, low-risk modality that has the capacity to augment treatment efficacy, especially in individuals experiencing partial remission, residual symptomatic burden, or concomitant metabolic and neurodevelopmental disorders. In the context of milder depressive manifestations, organized physical activity may function as an efficacious standalone intervention, particularly when implemented within educational or community frameworks. From a clinical integration standpoint, physical activity is increasingly acknowledged as an essential integrated component of multidisciplinary psychiatric care. Its incorporation into therapeutic regimens necessitates collaboration among psychiatrists, psychologists, exercise physiologists, and health professionals within educational settings. Importantly, the clinical positioning of exercise should be differentiated across evidence tiers, where some applications are strongly supported by interventional trials (e.g., adjunctive symptom reduction), whereas others remain conceptually grounded (e.g., standalone replacement therapy in moderate-to-severe depression), particularly in adolescent populations where direct comparative evidence is still limited. In this context, exercise should be conceptualized not as an isolated prescription, but as part of an integrated therapeutic framework in which psychological care, behavioral support, and social systems interact to enhance feasibility and engagement. The structured integration of physical activity within mental health services can still promote individualized dosing, facilitate adherence monitoring, and ensure alignment with patient preferences and functional abilities. Moreover, the amalgamation of exercise with digital mental health interventions or cognitive-behavioral approaches may augment patient engagement and therapeutic efficacy, signifying a transition towards multimodal and personalized intervention frameworks. At the policy level, the ramifications are of considerable importance. School-based physical activity initiatives constitute a scalable and economically viable strategy for population-level prevention and early intervention pertaining to adolescent mental health. Public health initiatives that emphasize the development of physical activity infrastructure, mitigation of participation barriers, and integration of mental health promotion into educational curricula may support adolescent mental health at a population level; however, these implications should be interpreted as emerging rather than definitive policy conclusions. Furthermore, recognizing exercise as an evidence-informed therapeutic component within integrated care models may help shape clinical guidelines and support its structured implementation within mental health systems, rather than positioning it solely as a lifestyle recommendation. In sum, these translational advancements underscore the necessity to reframe exercise not merely as a lifestyle suggestion, but as an essential element of evidence-informed adolescent mental health care.

An essential translational consideration is that the interplay between physical activity and adolescent depression is intrinsically bidirectional. Although engagement in physical exercise may ameliorate depressive symptoms through neurobiological, psychological, and behavioral pathways, the fundamental manifestations of depression encompassing anhedonia, fatigue, diminished motivation, reduced self-efficacy, social withdrawal, and negative body image, may concurrently hinder an adolescent’s capacity to initiate and maintain participation in exercise. Prior research has established that the severity of depressive symptoms is inversely related to levels of physical activity and adherence to exercise regimens among adolescents (86, 87). As a result, exercise should not be regarded as a universally applicable intervention that can be prescribed without regard to psychological context. Instead, its therapeutic efficacy may be contingent upon an individual’s psychological preparedness, emotional resources, familial environment, and systems of social support. These considerations are highly aligned with precision psychiatry frameworks, which emphasize individualized, context-sensitive treatment approaches incorporating biological, psychological, and social determinants of health. Within this paradigm, individualized exercise prescriptions ought to encompass not only exercise intensity, frequency, and biological indicators but also motivational status, self-efficacy, perceived obstacles, and emotional functioning (88). Empirical evidence from the field of behavioral medicine indicates that self-efficacy and motivation serve as critical predictors of sustained adherence to physical activity interventions, especially among individuals exhibiting depressive symptoms (89). Moreover, the integration of exercise programs with psychological interventions such as cognitive-behavioral therapy, motivational interviewing, or digital behavioral coaching, has been demonstrated to augment adherence rates and enhance mental health outcomes (43). Consequently, such integrative strategies may further enhance the feasibility, sustainability, and clinical effectiveness of exercise-based interventions when delivered within coordinated, multidisciplinary care systems.

## 7 Future directions

Future inquiries into exercise-based interventions aimed at ameliorating adolescent depression ought to transcend the assessment of general efficacy and advance towards more nuanced, mechanism-informed, and individualized methodologies. A significant progression is anticipated in the formulation of precision exercise prescriptions, wherein parameters such as intensity, duration, modality, and frequency are meticulously customized to align with individual biological, psychological, and environmental characteristics. Prevailing evidence indicates that variations in individual responses to exercise may be modulated by genetic determinants, initial fitness levels, severity of symptoms, and psychosocial circumstances. The incorporation of these moderating factors into clinical decision-making processes could substantially enhance therapeutic efficacy and optimize mental health outcomes. Another pivotal avenue of inquiry pertains to the imperative for long-term and mechanistic investigations. Although existing randomized controlled trials elucidate immediate advantages of physical exercise on depressive manifestations, anxiety, and stress, there exists an inadequate comprehension of enduring effects and mechanisms of relapse prevention. Longitudinal studies are crucial to ascertain whether exercise-induced enhancements in neuroplasticity, stress modulation, and emotional regulation are sustained over extended periods. Furthermore, a greater number of mechanistic investigations are warranted to elucidate the causal mechanisms that connect physical activity with cerebral function, particularly concerning BDNF signaling, regulation of the HPA axis, myokine-mediated communication between muscle and brain, and processes of epigenetic remodeling. Ultimately, the amalgamation of physical activity interventions with digital therapeutics signifies an exceptionally promising domain. Mobile health applications, wearable technology, and online behavioral platforms possess the capacity to enhance monitoring, feedback, and compliance, while simultaneously facilitating the real-time customization of exercise regimens. Furthermore, digital instruments may promote extensive implementation within educational and communal environments, thereby bridging the divide between controlled clinical investigations and practical, real-world applications. The intersection of exercise science, digital health innovations, and precision psychiatry harbors the potential to transform preventive and therapeutic paradigms for adolescent depression, ultimately fostering scalable, accessible, and biologically-informed mental health interventions.

## 8 Conclusions

The burgeoning corpus of evidence highlights exercise as a potentially beneficial and multifaceted intervention for the enhancement of mental health outcomes among adolescents, particularly in the mitigation of depressive symptoms and related comorbid conditions. In a spectrum of randomized controlled trials, structured physical activity, especially when administered at moderate-to-vigorous intensity or in conjunction with behavioral and educational components, has been associated with improvements in emotional well-being, stress management, and cognitive functioning. Significantly, these effects extend beyond symptom alleviation, encompassing advancements in resilience, quality of life, and functional capacity, all of which are essential for sustainable developmental trajectories. From a mechanistic standpoint, exercise may exert its antidepressant effects through multiple interrelated biological pathways, including the upregulation of neurotrophic factors such as BDNF, modulation of HPA axis function, reduction of oxidative stress, and induction of anti-inflammatory and myokine-mediated signaling. Collectively, these adaptations are thought to foster enhanced neuroplasticity and emotional regulation during a pivotal phase of cerebral maturation. Nonetheless, the heterogeneity in findings across various investigations underscores the necessity of accounting for individual variances, which encompass initial mental health conditions, levels of physical fitness, genetic susceptibility, and psychosocial environments. Furthermore, obstacles pertaining to adherence to interventions and their sustainability persist as significant challenges, particularly among adolescents with clinical disorders. The integration of digital mental health platforms, school-based interventions, and tailored exercise prescriptions presents a promising but still emerging approach to improving engagement and scalability. Prospective inquiries should concentrate on precision-oriented methodologies that aim to customize the type, intensity, and duration of exercise according to individual profiles, while further clarifying dose–response dynamics and long-term effects. However, it should be acknowledged that the current evidence base for fully operational precision psychiatry applications in exercise-based interventions remains preliminary and requires further validation. In summary, physical exercise constitutes a safe, accessible, and biologically informed approach that may serve as a promising preventive and complementary intervention for adolescent depression, warranting further rigorous investigation and thoughtful integration into clinical and public health frameworks.

## References

[1] Aarons GA Seijo C Green AE Moullin JC Hasson H von Thiele Schwarz U . Fostering international collaboration in implementation science and research: a concept mapping exploratory study. BMC Res Notes. (2019) 12:778. doi: 10.1186/s13104-019-4800-4 31783912 PMC6883596

[2] Guthold R Carvajal-Velez L Adebayo E Azzopardi P Baltag V Dastgiri S . The importance of mental health measurement to improve global adolescent health. J Adolesc Health. (2023) 72:S3–s6. doi: 10.1016/j.jadohealth.2021.03.030 36229397

[3] Smith AR Chein J Steinberg L . Impact of socio-emotional context, brain development, and pubertal maturation on adolescent risk-taking. Horm Behav. (2013) 64:323–32. doi: 10.1016/j.yhbeh.2013.03.006 23998675 PMC3761223

[4] Patel V Flisher AJ Hetrick S McGorry P . Mental health of young people: a global public-health challenge. Lancet. (2007) 369:1302–13. doi: 10.1016/s0140-6736(07)60368-7 17434406

[5] Cipriani A Zhou X Del Giovane C Hetrick SE Qin B Whittington C . Comparative efficacy and tolerability of antidepressants for major depressive disorder in children and adolescents: a network meta-analysis. Lancet. (2016) 388:881–90. doi: 10.1016/s0140-6736(16)30385-3 27289172

[6] Bridge JA Iyengar S Salary CB Barbe RP Birmaher B Pincus HA . Clinical response and risk for reported suicidal ideation and suicide attempts in pediatric antidepressant treatment: a meta-analysis of randomized controlled trials. Jama. (2007) 297:1683–96. doi: 10.1001/jama.297.15.1683 17440145

[7] Duman RS Aghajanian GK . Synaptic dysfunction in depression: potential therapeutic targets. Science. (2012) 338:68–72. doi: 10.1126/science.1222939 23042884 PMC4424898

[8] Beck A Nadkarni A Calam R Naeem F Husain N . Increasing access to cognitive behaviour therapy in low and middle income countries: a strategic framework. Asian J Psychiatry. (2016) 22:190–5. doi: 10.1016/j.ajp.2015.10.008 26643366

[9] Ricci M Pozzi G Caraglia N Chieffo DPR Polese D Galiuto L . Psychological distress affects performance during exercise-based cardiac rehabilitation. Life (Basel Switzerland). (2024) 14. doi: 10.3390/life14020236 38398745 PMC10890595

[10] Polese D . Beyond the heart: mind-body connection from birth and the holistic intervention in cardiovascular care. Int J Cardiol. (2025) 432:133270. doi: 10.1016/j.ijcard.2025.133270 40252848

[11] Erickson KI Hillman C Stillman CM Ballard RM Bloodgood B Conroy DE . Physical activity, cognition, and brain outcomes: a review of the 2018 physical activity guidelines. Med Sci Sports Exerc. (2019) 51:1242–51. doi: 10.1249/mss.0000000000001936 31095081 PMC6527141

[12] Cotman CW Berchtold NC . Exercise: a behavioral intervention to enhance brain health and plasticity. Trends Neurosci. (2002) 25:295–301. doi: 10.1016/s0166-2236(02)02143-4 12086747

[13] Spear LP . Adolescent neurodevelopment. J Adolesc Health. (2013) 52:S7–13. doi: 10.1016/j.jadohealth.2012.05.006 23332574 PMC3982854

[14] Wegner M Helmich I MaChado S Nardi AE Arias-Carrion O Budde H . Effects of exercise on anxiety and depression disorders: review of meta- analyses and neurobiological mechanisms. CNS Neurol Disord Drug Targets. (2014) 13:1002–14. doi: 10.2174/1871527313666140612102841 24923346

[15] Teixeira PJ Carraça EV Markland D Silva MN Ryan RM . Exercise, physical activity, and self-determination theory: a systematic review. Int J Behav Nutr Phys Act. (2012) 9:78. doi: 10.1186/1479-5868-9-78 22726453 PMC3441783

[16] McAuley E Blissmer B . Self-efficacy determinants and consequences of physical activity. Exerc Sport Sci Rev. (2000) 28:85–8. 10902091

[17] Plotnikoff RC Costigan SA Karunamuni N Lubans DR . Social cognitive theories used to explain physical activity behavior in adolescents: a systematic review and meta-analysis. Prev Med. (2013) 56:245–53. doi: 10.1016/j.ypmed.2013.01.013 23370047

[18] K BO Smith J Lubans DR Ng JY Lonsdale C . Self-determined motivation and physical activity in children and adolescents: a systematic review and meta-analysis. Prev Med. (2014) 67:270–9. doi: 10.1016/j.ypmed.2014.07.033 25073077

[19] Polanczyk GV Salum GA Sugaya LS Caye A Rohde LA . Annual research review: a meta-analysis of the worldwide prevalence of mental disorders in children and adolescents. J Child Psychol Psychiatry Allied Disciplines. (2015) 56:345–65. doi: 10.1111/jcpp.12381 25649325

[20] Thapar A Collishaw S Pine DS Thapar AK . Depression in adolescence. Lancet. (2012) 379:1056–67. doi: 10.1016/s0140-6736(11)60871-4 22305766 PMC3488279

[21] Salk RH Hyde JS Abramson LY . Gender differences in depression in representative national samples: meta-analyses of diagnoses and symptoms. psychol Bull. (2017) 143:783–22. doi: 10.1037/bul0000102 28447828 PMC5532074

[22] Felitti VJ Anda RF Nordenberg D Williamson DF Spitz AM Edwards V . Relationship of childhood abuse and household dysfunction to many of the leading causes of death in adults. The Adverse Childhood Experiences (ACE) Study. Am J Prev Med. (1998) 14:245–58. doi: 10.1016/j.amepre.2019.04.001 9635069

[23] Miller AH Raison CL . The role of inflammation in depression: from evolutionary imperative to modern treatment target. Nat Rev Immunol. (2016) 16:22–34. doi: 10.1038/nri.2015.5 26711676 PMC5542678

[24] Herbert TB Cohen S . Depression and immunity: a meta-analytic review. psychol Bull. (1993) 113:472–86. doi: 10.1037/0033-2909.113.3.472 8316610

[25] Gleeson M Bishop NC Stensel DJ Lindley MR Mastana SS Nimmo MA . The anti-inflammatory effects of exercise: mechanisms and implications for the prevention and treatment of disease. Nat Rev Immunol. (2011) 11:607–15. doi: 10.1038/nri3041 21818123

[26] Erickson KI Voss MW Prakash RS Basak C Szabo A Chaddock L . Exercise training increases size of hippocampus and improves memory. Proc Natl Acad Sci USA. (2011) 108:3017–22. doi: 10.1073/pnas.1015950108 21282661 PMC3041121

[27] Meinzer MC Pettit JW Viswesvaran C . The co-occurrence of attention-deficit/hyperactivity disorder and unipolar depression in children and adolescents: a meta-analytic review. Clin Psychol Rev. (2014) 34:595–607. doi: 10.1016/j.cpr.2014.10.002 25455624

[28] Xie Y Gao X Song Y Zhu X Chen M Yang L . Effectiveness of physical activity intervention on ADHD symptoms: a systematic review and meta-analysis. Front Psychiatry. (2021) 12:706625. doi: 10.3389/fpsyt.2021.706625 34764893 PMC8575983

[29] Luppino FS de Wit LM Bouvy PF Stijnen T Cuijpers P Penninx BW . Overweight, obesity, and depression: a systematic review and meta-analysis of longitudinal studies. Arch Gen Psychiatry. (2010) 67:220–9. doi: 10.1001/archgenpsychiatry.2010.2 20194822

[30] Pereira SS Alvarez-Leite JI . Low-grade inflammation, obesity, and diabetes. Curr Obes Rep. (2014) 3:422–31. doi: 10.1007/s13679-014-0124-9 26626919

[31] Li JR Tsai SJ Bai YM Hsu JW Huang KL Su TP . Cardiometabolic disease risk among siblings of patients with major depressive disorder. J Dev Orig Health Dis. (2021) 12:530–5. doi: 10.1017/s2040174420000860 32924904

[32] Sheeber L Hops H Davis B . Family processes in adolescent depression. Clin Child Family Psychol Rev. (2001) 4:19–35. doi: 10.1023/a:1009524626436 11388562

[33] Arseneault L . The long-term impact of bullying victimization on mental health. World Psychiatry: Off J World Psychiatr Assoc (WPA). (2017) 16:27–8. doi: 10.1002/wps.20399 28127927 PMC5269482

[34] Blanchard L Conway-Moore K Aguiar A Önal F Rutter H Helleve A . Associations between social media, adolescent mental health, and diet: a systematic review. Obes Rev. (2023) 24 Suppl 2:e13631. doi: 10.1111/obr.13631 37753597

[35] Daryabeygi-Khotbehsara R Shariful Islam SM Dunstan D McVicar J Abdelrazek M Maddison R . Smartphone-based interventions to reduce sedentary behavior and promote physical activity using integrated dynamic models: systematic review. J Med Internet Res. (2021) 23:e26315. doi: 10.2196/26315 34515637 PMC8477296

[36] Reiss F . Socioeconomic inequalities and mental health problems in children and adolescents: a systematic review. Soc Sci Med. (2013) 90:24–31. doi: 10.1016/j.socscimed.2013.04.026 23746605

[37] McGuinness TM Dyer JG Wade EH . Gender differences in adolescent depression. J Psychosocial Nurs Ment Health Serv. (2012) 50:17–20. doi: 10.3928/02793695-20121107-04 23457713

[38] Schuch FB Vancampfort D Firth J Rosenbaum S Ward PB Silva ES . Physical activity and incident depression: a meta-analysis of prospective cohort studies. Am J Psychiatry. (2018) 175:631–48. doi: 10.1176/appi.ajp.2018.17111194 29690792

[39] Guan J Sun Y Fan Y Liang J Liu C Yu H . Effects and neural mechanisms of different physical activity on major depressive disorder based on cerebral multimodality monitoring: a narrative review. Front Hum Neurosci. (2024) 18:1406670. doi: 10.3389/fnhum.2024.1406670 39188405 PMC11345241

[40] Collins H Booth JN Duncan A Fawkner S Niven A . The effect of resistance training interventions on 'the self' in youth: a systematic review and meta-analysis. Sports Med - Open. (2019) 5:29. doi: 10.1186/s40798-019-0205-0 31270635 PMC6609926

[41] Logan GR Harris N Duncan S Schofield G . A review of adolescent high-intensity interval training. Sports Med. (2014) 44:1071–85. doi: 10.1007/s40279-014-0187-5 24743929

[42] Lazzarelli A Scafuto F Crescentini C Matiz A Orrù G Ciacchini R . Interoceptive ability and emotion regulation in mind-body interventions: an integrative review. Behav Sci (Basel Switzerland). (2024) 14. doi: 10.3390/bs14111107 39594407 PMC11591285

[43] Ross RE VanDerwerker CJ Saladin ME Gregory CM . The role of exercise in the treatment of depression: biological underpinnings and clinical outcomes. Mol Psychiatry. (2023) 28:298–328. doi: 10.1038/s41380-022-01819-w 36253441 PMC9969795

[44] Jemni M Zaman R Carrick FR Clarke ND Marina M Bottoms L . Exercise improves depression through positive modulation of brain-derived neurotrophic factor (BDNF). A review based on 100 manuscripts over 20 years. Front Physiol. (2023) 14:1102526. doi: 10.3389/fphys.2023.1102526 36969600 PMC10030936

[45] Carter T Morres ID Meade O Callaghan P . The effect of exercise on depressive symptoms in adolescents: a systematic review and meta-analysis. J Am Acad Child Adolesc Psychiatry. (2016) 55:580–90. doi: 10.1016/j.jaac.2016.04.016 27343885

[46] Patikas DA Williams CA Ratel S . Exercise-induced fatigue in young people: advances and future perspectives. Eur J Appl Physiol. (2018) 118:899–910. doi: 10.1007/s00421-018-3823-1 29441401

[47] Biddle SJ Asare M . Physical activity and mental health in children and adolescents: a review of reviews. Br J Sports Med. (2011) 45:886–95. doi: 10.1136/bjsports-2011-090185 21807669

[48] Bailey AP Hetrick SE Rosenbaum S Purcell R Parker AG . Treating depression with physical activity in adolescents and young adults: a systematic review and meta-analysis of randomised controlled trials. Psychol Med. (2018) 48:1068–83. doi: 10.1017/s0033291717002653 28994355

[49] Schuch FB Vancampfort D Richards J Rosenbaum S Ward PB Stubbs B . Exercise as a treatment for depression: a meta-analysis adjusting for publication bias. J Psychiatr Res. (2016) 77:42–51. doi: 10.1016/j.jpsychires.2016.02.023 26978184

[50] Biddle SJ Ciaccioni S Thomas G Vergeer I . Physical activity and mental health in children and adolescents: An updated review of reviews and an analysis of causality. Psychol Sport Exercise. (2019) 42:146–55. doi: 10.1016/j.psychsport.2018.08.011 38826717

[51] Fu Q Li L Li Q Wang J . The effects of physical activity on the mental health of typically developing children and adolescents: A systematic review and meta-analysis. BMC Public Health. (2025) 25:1514. doi: 10.1186/s12889-025-22690-8 40269876 PMC12016293

[52] Fernandes BM Siqueira CC Vieira RM Moreno RA Soeiro-de-Souza MG . Physical activity as an adjuvant therapy for depression and influence on peripheral inflammatory markers: A randomized clinical trial. Ment Health Phys Activity. (2022) 22:100442. doi: 10.1016/j.mhpa.2022.100442 38826717

[53] Cooney GM Dwan K Greig CA Lawlor DA Rimer J Waugh FR . Exercise for depression. Cochrane Database Syst Rev. (2013) 2013:Cd004366. doi: 10.1111/jebm.12076 24026850 PMC9721454

[54] Chen L Zhao G Shi X . Digital mental health literacy combined with physical activity for depression and anxiety in high school students: A randomized controlled trial. J School Health. (2026) 96:e70144. doi: 10.1111/josh.70144 41957996

[55] Chang Q Han X Shen W Yang B Lin Z . Effects of varying exercise intensities on muscle strength and depressive symptoms in Chinese adolescents: A 12-week randomized controlled trial. PloS One. (2025) 20:e0336894. doi: 10.1371/journal.pone.0336894 41270072 PMC12637978

[56] Dastamooz S Wong SHS Yang Y Arbour-Nicitopoulos K Ho RTH Yam JCS . Efficacy of a short-term physical exercise intervention on stress biomarkers and mental health in adolescents with ADHD: A randomized controlled trial. J Affect Disord. (2026) 393:120285. doi: 10.1016/j.jad.2025.120285 40945770

[57] Li Q Zhang M Cai Y Zhuang S . Effect of 6-week moderate- and low-intensity exercise and individualized nutrition interventions on the quality of life in obese, hypertensive adolescents. Endocrine. (2025) 90:579–87. doi: 10.1007/s12020-025-04370-0 40828302

[58] Liu C Yang Y Wong SH Leung A Sit CH . The effects of physical activity on mental health in adolescents with attention-deficit hyperactivity disorder: A randomized controlled trial. Int J Behav Nutr Phys Act. (2025) 22:47. doi: 10.1186/s12966-025-01745-4 40247314 PMC12007287

[59] Huang H Xiao X . A randomized controlled trial of a combined physical activity and attitude education intervention on weight stigma and health outcomes. J School Health. (2025) 95:341–8. doi: 10.1111/josh.70000 39935336

[60] da Silva JM Castilho Dos Santos G de Oliveira Barbosa R de Souza Silva TM Correa RC da Costa BGG . Effects of a school-based physical activity intervention on mental health indicators in a sample of Brazilian adolescents: A cluster randomized controlled trial. BMC Public Health. (2025) 25:539. doi: 10.1186/s12889-025-21620-y 39930438 PMC11809091

[61] Mayer JS Kohlhas L Stermann J Medda J Brandt GA Grimm O . Bright light therapy versus physical exercise to prevent co-occurring depression in adolescents and young adults with attention-deficit/hyperactivity disorder: A multicentre, three-arm, randomised controlled, pilot phase-IIa trial. Eur Arch Psychiatry Clin Neurosci. (2025) 275:653–65. doi: 10.1007/s00406-024-01784-1 38627266 PMC11946981

[62] Ahmed KR Horwood S Kolbe-Alexander T Khan A . Effects of a school-based physical activity intervention on adolescents' mental health: A cluster randomized controlled trial. J Phys Act Health. (2023) 20:1102–8. doi: 10.1123/jpah.2023-0062 37611913

[63] Melnyk BM Jacobson D Kelly SA Belyea MJ Shaibi GQ Small L . Twelve-month effects of the COPE Healthy Lifestyles TEEN Program on overweight and depressive symptoms in high school adolescents. J School Health. (2015) 85:861–70. doi: 10.1111/josh.12342 26522175 PMC5117907

[64] Carter T Guo B Turner D Morres I Khalil E Brighton E . Preferred intensity exercise for adolescents receiving treatment for depression: A pragmatic randomised controlled trial. BMC Psychiatry. (2015) 15:247. doi: 10.1186/s12888-015-0638-z 26467764 PMC4605143

[65] Carter T Morres I Repper J Callaghan P . Exercise for adolescents with depression: Valued aspects and perceived change. J Psychiatr Ment Health Nurs. (2016) 23:37–44. doi: 10.1111/jpm.12261 26289604

[66] Dulfer K Duppen N Van Dijk AP Kuipers IM Van Domburg RT Verhulst FC . Parental mental health moderates the efficacy of exercise training on health-related quality of life in adolescents with congenital heart disease. Pediatr Cardiol. (2015) 36:33–40. doi: 10.1007/s00246-014-0961-z 25077662

[67] Melnyk BM Jacobson D Kelly S O'Haver J Small L Mays MZ . Improving the mental health, healthy lifestyle choices, and physical health of Hispanic adolescents: A randomized controlled pilot study. J School Health. (2009) 79:575–84. doi: 10.1111/j.1746-1561.2009.00451.x 19909421

[68] Daley AJ Copeland RJ Wright NP Roalfe A Wales JK . Exercise therapy as a treatment for psychopathologic conditions in obese and morbidly obese adolescents: A randomized, controlled trial. Pediatrics. (2006) 118:2126–34. doi: 10.1542/peds.2006-1285 17079587

[69] Crews DJ LM Landers DM . Aerobic physical activity effects on psychological well-being in low-income Hispanic children. Percept Mot Skills. (2004) 98:319–24. doi: 10.2466/pms.98.1.319-324 15058892

[70] Kanani J . Diagnostic challenges in hemangioblastoma: Lessons from a rare case presentation. Egyptian J Neurosurg. (2025) 40:115. doi: 10.1186/s41984-025-00461-2 38164791

[71] Gejl AK Bugge A Ernst MT Mortensen EL Gejl KD Andersen LB . Effects of 9 weeks of high- or moderate-intensity training on cardiorespiratory fitness, inhibitory control, and plasma brain-derived neurotrophic factor in Danish adolescents-a randomized controlled trial. Scandinavian J Med Sci Sports. (2024) 34:e14703. doi: 10.1111/sms.14703 39054765

[72] Williams RA Cooper SB Dring KJ Hatch L Morris JG Sunderland C . Effect of football activity and physical fitness on information processing, inhibitory control and working memory in adolescents. BMC Public Health. (2020) 20:1398. doi: 10.1186/s12889-020-09484-w 32928161 PMC7488749

[73] Roh HT Cho SY So WY . Effects of regular Taekwondo intervention on oxidative stress biomarkers and myokines in overweight and obese adolescents. Int J Environ Res Public Health. (2020) 17. doi: 10.3390/ijerph17072505 32268592 PMC7177505

[74] Walsh JJ D'Angiulli A Cameron JD Sigal RJ Kenny GP Holcik M . Changes in the brain-derived neurotrophic factor are associated with improvements in diabetes risk factors after exercise training in adolescents with obesity: The HEARTY randomized controlled trial. Neural Plast. (2018) 2018:7169583. doi: 10.1155/2018/7169583 30363954 PMC6186331

[75] Goldfield GS Kenny GP Prud'homme D Holcik M Alberga AS Fahnestock M . Effects of aerobic training, resistance training, or both on brain-derived neurotrophic factor in adolescents with obesity: The hearty randomized controlled trial. Physiol Behav. (2018) 191:138–45. doi: 10.1016/j.physbeh.2018.04.026 29679660

[76] Moreau D Kirk IJ Waldie KE . High-intensity training enhances executive function in children in a randomized, placebo-controlled trial. eLife. (2017) 6. doi: 10.7554/elife.25062 28825973 PMC5566451

[77] Plaza-Florido A Esteban-Cornejo I Mora-Gonzalez J Torres-Lopez LV Osuna-Prieto FJ Gil-Cosano JJ . Gene-exercise interaction on brain health in children with overweight/obesity: The ActiveBrains randomized controlled trial. J Appl Physiol (Bethesda Md 1985). (2023) 135:775–85. doi: 10.1152/japplphysiol.00435.2023 37589055 PMC10642513

[78] Ghafori R HA Aghadsi MT . Effect of motor exercises on serum level of brain-derived neurotrophic factor and executive function in children with dysgraphia. J Kermanshah Univ Med Sci. (2018) 22:e79187. doi: 10.5812/jkums.79187

[79] Kim HT SY Kang EB Cho JY Kim BW Kim CH . The effects of combined exercise on basic physical fitness, neurotrophic factors and working memory of elementary students. Exerc Sci. (2015) 24:243–51. doi: 10.15857/ksep.2015.24.3.243

[80] Cho SY So WY Roh HT . The effects of Taekwondo training on peripheral neuroplasticity-related growth factors, cerebral blood flow velocity, and cognitive functions in healthy children: A randomized controlled trial. Int J Environ Res Public Health. (2017) 14. doi: 10.3390/ijerph14050454 28441325 PMC5451905

[81] Domingues-Montanari S . Clinical and psychological effects of excessive screen time on children. J Paediatr Child Health. (2017) 53:333–8. doi: 10.1111/jpc.13462 28168778

[82] van Deursen AJ van Dijk JA . The first-level digital divide shifts from inequalities in physical access to inequalities in material access. New Media Soc. (2019) 21:354–75. doi: 10.1177/1461444818797082 30886536 PMC6380454

[83] Naresh VS Thamarai M . Privacy‐preserving data mining and machine learning in healthcare: Applications, challenges, and solutions. Wiley Interdiscip Reviews: Data Min Knowledge Discov. (2023) 13:e1490. doi: 10.1002/widm.1490 41531421

[84] Eysenbach G . The law of attrition. J Med Internet Res. (2005) 7:e11. doi: 10.2196/jmir.7.1.e11 15829473 PMC1550631

[85] Proctor EK Landsverk J Aarons G Chambers D Glisson C Mittman B . Implementation research in mental health services: An emerging science with conceptual, methodological, and training challenges. Administration Policy Ment Health. (2009) 36:24–34. doi: 10.1007/s10488-008-0197-4 19104929 PMC3808121

[86] Stubbs B Koyanagi A Schuch FB Firth J Rosenbaum S Veronese N . Physical activity and depression: A large cross‐sectional, population‐based study across 36 low‐and middle‐income countries. Acta Psychiatrica Scandinavica. (2016) 134:546–56. doi: 10.1111/acps.12654 27704532

[87] Jiang W Sun L He Y Liu W Hua Y Zheng X . Association between physical activity and depressive symptoms in adolescents: A prospective cohort study. Psychiatry Res. (2023) 329:115544. doi: 10.1016/j.psychres.2023.115544 37852160

[88] Arango C Vieta E Fañañás L Courtet P De Picker L Kas MJH . Precision medicine in mental health: Applications, challenges, and recommendations. Eur Psychiatry J Assoc Eur Psychiatrists. (2026) 69:e53. doi: 10.1192/j.eurpsy.2026.12210 42037368 PMC13199632

[89] Ntoumanis N Ng JYY Prestwich A Quested E Hancox JE Thøgersen-Ntoumani C . A meta-analysis of self-determination theory-informed intervention studies in the health domain: Effects on motivation, health behavior, physical, and psychological health. Health Psychol Rev. (2021) 15:214–44. doi: 10.1080/17437199.2020.1718529 31983293

